# Preoperative heart rate variability as a predictor of perioperative outcomes: a systematic review without meta-analysis

**DOI:** 10.1007/s10877-022-00819-z

**Published:** 2022-01-29

**Authors:** Mikkel Nicklas Frandsen, Jesper Mehlsen, Nicolai Bang Foss, Henrik Kehlet

**Affiliations:** 1 Section for Surgical Pathophysiology, Copenhagen University Hospital, Rigshospitalet, Copenhagen, Denmark; 2 Department of Anesthesiology and Intensive Care, Hvidovre University Hospital, Hvidovre, Denmark

**Keywords:** Heart rate variability, Hypotension, Anesthesia, Perioperative medicine, Perioperative monitoring, Surgical outcome

## Abstract

Heart rate variability (HRV) is a predictor of mortality and morbidity after non-lethal cardiac ischemia, but the relation between preoperatively measured HRV and intra- and postoperative complications is sparsely studied and most recently reviewed in 2007. We, therefore, reviewed the literature regarding HRV as a predictor for intra- and postoperative complications and outcomes. We carried out a systematic review without meta-analysis. A PICO model was set up, and we searched PubMed, EMBASE, and CENTRAL. The screening was done by one author, but all authors performed detailed review of the included studies. We present data from studies on intraoperative and postoperative complications, which were too heterogeneous to warrant formal meta-analysis, and we provide a pragmatic review of HRV indices to facilitate understanding our findings. The review was registered in PROSPERO (CRD42021230641). We screened 2337 records for eligibility. 131 records went on to full-text assessment, 63 were included. In frequency analysis of HRV, low frequency to high frequency ratio could be a predictor for intraoperative hypotension in spinal anesthesia and lower total power could possibly predict intraoperative hypotension under general anesthesia. Detrended fluctuation analysis of HRV is a promising candidate for predicting postoperative atrial fibrillation. This updated review of the relation between preoperative HRV and surgical outcome suggests a clinically relevant role of HRV but calls for high quality studies due to methodological heterogeneity in the current literature. Areas for future research are suggested.

**Supplementary Information:**

The online version contains supplementary material available at 10.1007/s10877-022-00819-z.

## Introduction

Heart rate variability (HRV) is an index of neurocardiac function and is mediated primarily by sympathetic and vagal innervation of the heart [1]. As such, it may provide information about preexisting comorbidity and physiological status, as well as dynamic observation of physiological interaction with perioperative events. HRV is derived from an ECG of a patient in sinus rhythm. The length of the ECG sampling varies between studies, ranging from seconds to weeks. When reading such studies, it is important to bear in mind that studies of differing length cannot be compared as longer recordings include variability from a larger number of physiological systems [1]. Noise in the ECG signal in form of arrhythmias and ectopic beats presents a challenge, and these beats should be removed for classical HRV analysis. This can be done manually by inspecting the raw ECG signal, by software, or a combination of the two [2].

HRV has been suggested to be a predictor of mortality, both from cardiac causes such as arrhythmia and in patients after myocardial infarction in non-surgical populations [3–5]. Subsequently, Kawamoto et al. [6] observed that hypotension and bradycardia during spinal anesthesia (SA) were associated with concomitant changes in HRV associated with the induced sympatholysis caused by the neuroaxial blockade. Laitio et al. reviewed the literature and suggested that HRV indices could predict prolonged postsurgical stay in the intensive care unit (ICU) and mortality [7].

In this review, we update the current knowledge on the use of preoperative (preOP) HRV measurements as a predictor for intra- (IntraOP) and postoperative (postOP) outcomes, specifically regarding cardiovascular complications, mortality, and length of stay (LOS). For the purpose of this study, we defined the intraOP period as the time between induction of anesthesia and the end of the surgical procedure.

To facilitate interpretation of the used HRV methodology we briefly review the used indices of HRV from the three major domains of analysis below: Time, frequency, and nonlinear methods. For an in-depth review of the HRV methodology we refer to specialized literature [8, 9]. We also supply supplementary tables relating HRV indices to complications and vice versa (Supplementary Tables 1, 2, 3 and 4) in both SA and general anesthesia (GA).

### Time domain analysis

Overall HRV can be expressed by the standard deviation of normal to normal intervals (NNI), that is, adjacent R-peaks in the ECG during sinus rhythm—standard deviation of normal to normal interval (SDNN). SDNN is calculated over the whole recording, be it 5 min or multiple days. Overall variability can also be expressed by the triangular index as the frequency histogram of groups of NNI resembles a triangle. The index is expressed by the total number of NNI divided by the number of NNI in the most frequent NN-interval group. Short term variation in heart rate is quantified by the mean numerical difference between adjacent NNI which—in mathematical terms—is obtained as the square root of the mean of the successive squared differences in NNI over the whole recording—rMSSD. pNN50 is the percentage of successive beats differing by more than 50 ms [8]. The analgesia-nociception index is a normalized measure of the impact of respiration on HRV, closely related to the high frequency analysis presented below [10].

### Frequency domain analysis

HRV can be analyzed in the frequency domain by frequency analysis of the heart rate signal where the overall HRV is expressed by the total power (TP). These analyses can be done by various methods,^1^ potentially causing different results making comparison between methods difficult [2]. Certain frequency bands can be identified and related to specific control systems. Variability in the high frequency (HF) band (0.15–0.40 Hz) is closely related to respiration and activity in myelinated vagal nerve fibers, whereas variability in the low frequency (LF) band (0.04–0.15 Hz) is dominated by baroreceptor regulation and activity in a mix of sympathetic and parasympathetic nerve fibers [11]. These measures can be “normalized” through division by TP in order to explain how much of the total variation takes place in that specific frequency band. The very low frequency (VLF) band (0.0033–0.04 Hz) is less well-defined physiologically but can in free moving patients be related to physical activity [1]. The LF/HF ratio has previously been related to the balance of activity between the two main sections of the autonomic nervous system, but may more correctly be seen as the relation between the baroreflex and the parasympathetic modulation of cardiovascular control [8, 11].

### Nonlinear methods

Nonlinear methods aim to describe the predictability, chaos, self-similarity, and complexity of the HRV. The Poincaré plot shows each NNI plotted against the preceding NNI and is thus a graphic illustration of the predictability of successive heart beats. The point distribution in the plot resembles an ellipse and the standard deviation in the short axis (SD1) reflects short term variation whereas the standard deviation in the long axis (SD2) reflects long term variation. Tau is an expression of the correlation of one NNI to the next one, a measure of linearity. Prediction error is another way of determining the predictability of a heat rate series, in which a computer is trained on half of the dataset and then used to predict the second half of the set, quantifying the difference between the original and the predicted dataset. The Lyapunov exponent quantifies the amount of chaos, with higher values indicating chaos, and lower values indicating predictability. Fractal dimension is a measure of the complexity and self-similarity of the signal. Hurst exponent and detrended fluctuation analysis (DFA) are ways to describe this. Like the Poincare plot, the DFA can be divided into a short-term (DFA α1) and a long-term measure (DFA α2) [9, 12]. Correlation dimension (D2) and the associated measure, peak point correlation dimension (pPD2) are other ways of describing the fractal dimension. Complexity can be described by measures of entropy with higher values indicate more randomness of the system, and lower values indicating more regularity [9, 12]. While the exact algorithm for the ANSindex measure is proprietary, it is supposedly a measure of the sympatho-vagal balance by analyzing self-similarity and continuity of the RR-interval time series [13]. Symbolic dynamic analysis requires translation of the NNI to one of four nominal symbols (0 to 3) depending on whether there is a large or small increase or decrease. Three of these symbols then form “words” with 64 possible combinations of which “Forbidden Words” are rare symbol combinations. A lower count indicates higher complexity of the signal [14].

### Dynamic provocation tests

There are multiple other ways to evaluate the autonomic nervous system than baseline HRV. Several of the reviewed studies utilized dynamic testing with the Valsalva maneuver, positional changes, blood pressure monitoring, and a forced breathing protocol to assess autonomic response to circulatory challenges [15–17]. Also, Ackland and Abbot et al. have demonstrated decreased heart rate recovery after exercise testing to be associated with increased morbidity after noncardiac surgery [18, 19]. While the results of these provocation tests are physiologically interesting, we, however, chose to review resting baseline HRV measured by ECG, as this is the simplest and is most easily translated into clinical practice with minimal requirement for personnel training and acquisition of equipment.

## Methods

### Design

This study uses a systematic review approach to data searching and reporting, but meta-analysis was not performed due to heterogeneity of the included studies. One author performed the initial screening of title and abstracts, while detailed review of records was done by the four authors.

### Eligibility criteria

Based upon the above, we set up the following criteria for eligibility of published studies.

Inclusion criteria:

- PreOP resting measurement of HRV, within 1 week before operation
- Reported intraOP and/or postOP outcome measures related to HRV within 30 days postOP
- Human studies

Exclusion criteria:

- Pooling of data on operated and non-operated patients
- Pooling of pre- and postOP HRV data
- No outcome data available

### Search strategy

A search matrix was set up, inspired by the PICO model [20], but modified (Table 1).

**Table 1:** Search matrix

Investigation	Time	Outcome
“Heart rate variability”	Preoperative	Morbidity
HRV	Postoperative	Mortality
“Heartrate variability”	Perioperative	Adverse event
“Heart-rate variability”	Surgery	Adverse events
“Heart rate variation”	Presurgical	Complication
	Postsurgical	Complications
	Pre-operative	Survival
	Post-operative	Recovery
	Spinal anesthesia	Hypotension
	General anesthesia	Outcome
	Local anesthesia	Bradycardia
	Subarachnoid block	Pain
		Infection

Using the Boolean operators “AND” between columns, and “OR” between rows in each column, we yielded the following search string:

*("Heart rate variability" OR HRV OR "heartrate variability" OR "heart-rate variability" OR "Heart rate variation") AND (Preoperative OR pre-operative OR postoperative OR post-operative OR perioperative OR surgery OR presurgical OR postsurgical OR spinal anesthesia OR General anesthesia OR local anesthesia OR Subarachnoid block) AND (Morbidity OR Mortality OR Adverse event OR Adverse events OR Complication OR Complications OR Survival OR Recovery OR hypotension OR outcome OR bradycardia OR pain OR infection).*

**Fig. 1:**
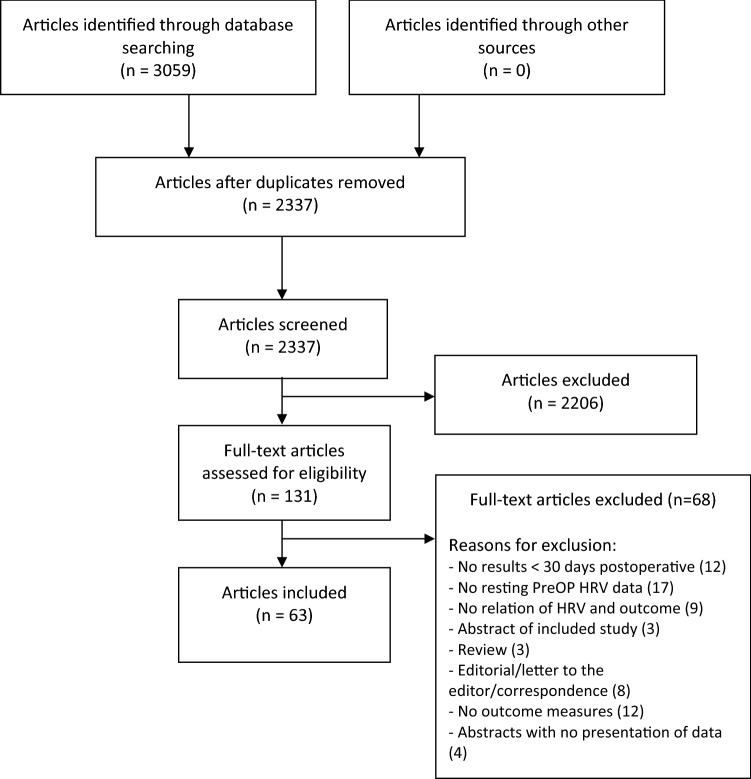
Flowchart depicting handling of records

Using this search string we searched PubMed, EMBASE, and CENTRAL, which as of 9/1-2021 returned 1221, 1509, and 329 records, totaling 3059. Figure 1 details the record screening process, using a PRISMA template [21]. To ensure optimal data capture we screened the quality of our search by cross-referencing the identified records, with the references in earlier reviews and from the records captured in our search. We found no relevant uncaptured records and as such, we were satisfied with our search terms. References were handled in Zotero 5.0.92. The protocol for this review was registered in PROSPERO (CRD42021230641).

## Results

### Spinal anesthesia

#### Hypotension

The most well researched area in prognostic preOP HRV is its relationship to the occurrence of intraOP hypotension with spinal SA, particularly in C-section (Table 2). All studies in this segment use lumbar SA with bupivacaine, except one. The definition of hypotension ranged from > 10 to 30% drop in mean arterial or systolic blood pressure or expressed in absolute terms as systolic blood pressure below 90–100 mmHg. Hanss et al. [22] retrospectively defined LF/HF higher than 2.5 as a predictor of hypotension and prospectively tested this hypothesis by including 19 women and assigning them to either high-LF/HF group (n = 9) or low-LF/HF (n = 10) based on data derived from their ECG the day before surgery. At baseline, there were no differences in hemodynamics but during the procedure, the high-LF/HF group had significantly lower nadirs in MAP (p < 0.05). The groups with moderate or severe hypotension also showed significantly higher LF/HF than the group with mild hypotension when measured in the operating room (OR) on the day of surgery (p < 0.05), whereas HRV measured the day before surgery did not reach statistical significance. Following this, Hanss et al. did an interventional study, on the use of prophylactic treatment guided by HRV. This study had an observational arm as well, where they saw significant differences in the occurrence of hypotension between groups divided by LF/HF = 2.5, with a higher incidence in the group with higher LF/HF, when measured in the OR on the day of surgery, but not the day before [23]. Bishop et al. [24] retrospectively analyzed 102 women undergoing C-section and found that the hypotensive parturients had higher LF/HF than their normotensive counterparts (p = 0.04) measured one hour before surgery. They defined a cut-off point of LF/HF > 2.0 to be a predictor of intraOP hypotension (p = 0.003). Prashanth et al. [25] evaluated the ANSindex measured on the day of surgery as a predictor of hypotension and found that for every one percentage-point increase in ANSindex, the risk of hypotension after SA for C-section increased by 8.3% (p = 0.002). Chamchad et al. [26] allocated 22 women undergoing C-section with SA into two groups of 11, separated by the median of pPD2 (3.9) measured in the OR immediately before surgery. This cut-off predicted the occurrence of intraOP hypotension, such that the 11 women with the lowest pPD2 all had hypotension, and none of the 11 with the highest pPD2 had hypotension. There was a tendency towards a difference in LF/HF between groups for the occurrence of subsequent hypotension (p = 0.07), with numerically higher values in those with intraOP hypotension during SA. Bolea et al. [17] identified significantly lower approximate entropy measured in the OR before spinal blockade for C-section as a predictor for hypotension (p = 0.03).

**Table 2:** Overview of studies on post-induction hypotension in C-section

Name and year	n =	Timing	Recording length	Measures that predicted hypotension
Hanss et al. (2005) [22]	60	DBS and DOS	5 min	LF/HF
				LF
				HF
Hanss et al. (2006) [23, 33]	40	DBS and DOS	5 min	LF/HF
Bishop et al. (2017) [24]	102	DOS	5 min	LF/HF
Chamchad et al. (2004) [26]	22	DOS	10 min	pPD2
Bolea et al. (2017) [17]	51	DOS	7 min	ApEn
Prashanth et al. (2017) [25]	108	DOS	8 min	ANSindex
Jendoubi et al. (2020) [28]	100	DOS	N/A	No predictors
Yokose et al. (2015) [27]	81	DOS	3 min	No predictors
Helmy Shehata et al. (2019) [29]	41	DOS	N/A	No predictors

*DOS* day of surgery, *DBS* day before surgery

In contrast, three studies found no difference between hypo- and normotensive parturients undergoing C-section when measuring SDNN, analgesia-nociception index, LF/HF, and entropy, all measured in the OR on the day of surgery [27–29].

In other types of surgeries, diabetic patients undergoing unspecified surgery using SA SDNN (p = 0.045), rMSSD (p = 0.032), and LF (p = 0.042) were all significantly lower the day before surgery in the group experiencing intraOP hypotension [30]. In unspecified infra-umbilical surgery with SA, Sharma et al. [31] allocated 100 patients into two groups based on their LF/HF being above or below 2.5 at preanesthetic check on the day of surgery. They found significantly lower intraOP blood pressure in the high-LF/HF group similar to some of the above-mentioned C-section studies. They also found a higher proportion of patients receiving vasopressors in the high vs. low LF/HF group. Raimondi et al. [32] grouped patients undergoing lower abdominal or lower limb surgery, by the median LF/HF (= 2.3) in the OR on the day of surgery. Significantly more patients in the group with higher LF/HF developed hypotension after SA. In prostate brachytherapy during SA, Hanss et al. [33] first retrospectively reviewed 30 patients to define a cut-off point of LF/HF = 2.5, then prospectively included 70 patients to test this cut-off. Again, they showed that those with values above the cut-off in the OR on the day of surgery had significantly lower nadirs in systolic blood pressure than those with values below (p < 0.05). However, a study in hypertensive patients undergoing orthopedic lower limb surgery found no significant predictive value for hypotension from TP or LF/HF when measured in the OR on the day of surgery [34]. In elderly patients undergoing plastic or orthopedic lower limb surgery hypotension did not occur more frequently in a group with higher LF/HF (> 2.5) in the OR on the day of surgery compared to those with lower values [35]. Fujiwara et al. allocated 52 patients undergoing transurethral surgery with SA, into two groups based on their ultra-short entropy, measured in the OR before surgery, being above or below the mean for the total group. There was a significantly higher incidence of hypotension in the group with low compared to high entropy. The group with low entropy also had significantly higher LF/HF but analysis of groups stratified by LF/HF was not reported [36]. This study only recorded 30 s ECG which is shorter than recommended [8].

The only study not using SA (thoracic surgery with high epidural anesthesia) found that an LF/HF ratio > 2.5 (p = 0.04) measured at an unspecified time on the day of surgery, but not other frequency domain indices were significantly correlated with hypotension [37].

To summarize, LF/HF fared best of the indices, with higher values being significantly predictive of hypotension in 8 of 13 studies when measured on the day of surgery, whereas there was no predictive power of HRV in the three studies measuring HRV on the day before surgery. A single study showing positive results only recorded 30-s ECG [36], and two studies did not specify recording length [30, 37]. The rest of the studies used comparable recording lengths (3–10 min), calling for standardized reporting of HRV assessment.

#### Bradycardia

A single study examined the relation of HRV on the day of surgery to bradycardia in patients receiving SA with bupivacaine for lower abdominal or lower limb surgery. They found higher preOP HF in the OR (p < 0.049), but similar LF and LF/HF in the bradycardic patients, defined by having a heart rate < 45 bpm during the procedure [38].

### General anesthesia

#### Hypotension

Two studies on hypotension under GA utilized total intravenous anesthesia [39, 40], while two did not specify the anesthesia [41, 42], and six received inhalational anesthesia. Induction of anesthesia was either with propofol [16, 39, 40, 43], etomidate [44, 45], thiopental [15, 46] or unspecified [41, 42]. Definition of hypotension ranged from 30 to 40% decrease in MAP from baseline, MAP < 60–80 mmHg or SBP < 80–90 mmHg. In abdominal surgery, Padley et al. [43] found multiple significant differences in HRV indices measured several days (IQR 1 to 11 days) before surgery. The measured indices comprised all three domains and the highest values in ROC analyses for predicting hypotension were SDNN, rMSSD, HF, D2, and Poincare SD1&2 (AUC > 0.85 and p < 0.003 for each of the measures). A study in diabetics undergoing ophthalmic surgery found that lower coefficient of variation, measured as the SDNN divided by the mean NNI, (p = 0.008), rMSSD (p = 0.022), and measurements close in frequency bands, but not identical to VLF, LF, and HF measured the day before surgery predicted hypotensive periods during induction of general anesthesia [46]. Huang et al. [45] found that lower HF and TP measured in the OR immediately before surgery were predictors for intraOP hypotension in unspecified surgery. However, their analysis of HRV as an independent predictor is limited by the fact that individuals experiencing hypotension were significantly older, had higher ASA scores, and included more diabetics compared to the group without hypotension. Hanss et al. [39] retrospectively reviewed 50 patients and identified TP less than 500ms^2^ measured on the day of surgery, to be a risk factor for hypotension in abdominal or vascular surgery in high risk cardiovascular patients (p = 0.04). Subsequently, in a prospective cohort, they found that TP measured on the day of surgery was the only HRV parameter that predicted lower nadirs in MAP (p < 0.05) [22, 33]. In abdominal surgery Reimer et al. [16] allocated patients according to preOP TP and orthostatic testing. The group with higher LF/HF and lower TP, LF, and HF prior to orthostatic testing on the day before surgery showed significantly increased need for rescue vasoactive drugs during surgery (p = 0.0002) and greater loss of blood (p = 0.0140), which could explain the need for more vasoactive drugs. A study by Latson et al. [15] showed that lower TP and lower HRV in frequency bands close to, but not identical to HF and LF on the day of surgery predicted hypotension in patients undergoing unspecified surgery (p < 0.009 for all four indices). However, the resting HRV was measured after other tests for autonomic dysfunction, and thus might not truly represent resting HRV. In spine surgery, Raghavan et al. found higher LF/HF (p < 0.001) and HF, but not TP, VLF or LF to be predictive of hypotension after induction, but did not specify when this was measured in the preOP phase [41]. Diabetic patients undergoing unspecified surgery, showed both lower TP (p < 0.003) on the day of surgery and an increased occurrence of hypotension (p < 0.04), compared to non-diabetic subjects, which could be expected, as diabetes is associated with lower HRV [42]. Fujiwara et al. [40] grouped patients according to preOP ultra-short entropy above or below 45 in the OR on the day of unspecified surgery, and found significantly more hypotensive patients in the group with lower entropy (p < 0.0001). Lower entropy correlated with lower LF and HF power as well as higher LF/HF ratio, but their relation to hypotension was not analyzed. Aiming to predict hypotension after tourniquet deflation in total knee arthroplasty, Huh et al. found no relation between HRV measured in the OR on the day of surgery and occurrence of hypotension [44].

Summarizing, preOP TP was lower in the hypotensive group in six of seven studies. Five studies assessed TP on the day of surgery, and four of these were with positive results. The remaining two measured on the day before, or several days before surgery and both predicted hypotension. Reduced HF was predictive in six of eight studies. Five studies assessed HF on the day of surgery, with three showing positive results. Two measured on the day before, and one, several days before surgery, all showing positive results. All studies used comparable lengths of ECG recording (5–15 min).

In conclusion, lower TP and HF on the day of surgery seem to predict intraOP hypotension under general anesthesia.

#### Bradycardia

One study used inhalational anesthetic [47] and two used intravenous technique [39, 48]. All used intravenous induction. Definitions of bradycardia differed between studies, ranging from a 20–40% fall in heart rate, or an absolute heart rate below 50 bpm. A study in pediatric strabismus surgery measured HRV on the day before surgery and found that reduced rMSSD (p < 0.005), pNN50 (p < 0.05), HF (p < 0.05), and nonlinear measures (prediction error (p < 0.005) and fractal dimension (p < 0.05)) predicted presence of oculocardiac reflex resulting in intra-operative bradycardia [47]. Estafanous et al. studied the occurrence of bradycardia in coronary artery bypass grafting (CABG), and found that HF was lower (p < 0.05) and LF/HF higher (p < 0.05) in the bradycardic group compared to the group with a stable heart rate, when measured in the OR before surgery [48]. Hanss et al. found that patients undergoing abdominal or vascular surgery with lower preOP TP (p < 0.05) on the day of surgery had a larger fall in heart rate after induction, but LF/HF was not predictive [39].

Summarizing, the best predictor for intraOP bradycardia seems to be a low HF, showing positive results in two of three studies.

### Postoperative pulmonary complications

Elwood et al. [49] found no significant differences in regular frequency domain measures between children with adverse respiratory events (‘wheezing’ and laryngospasm), when emerging from anesthesia after surgery. They did however find a lower ratio between standing and lying LF/HF (p = 0.019). Another study found reduced a preOP LF/HF to be predictive of pneumonia after hip fracture surgery but did not specify when this was measured [50]. Corrêa et al. [51] found that preOP nonlinear HRV analyses (lyapunov, approximate entropy, and DFA) were related to an increased risk of pulmonary infection after myocardial revascularization when measured the day before surgery. Heterogeneity between outcomes does not allow any collective conclusions.

### Pain

#### Intraoperative pain with local anesthesia

Two studies evaluated HRV on the day of surgery as a predictor for intraOP pain after varicose vein surgery. Patients received local tumescent anesthesia with lidocaine and epinephrine before the procedure. The authors then incorporated HF, LF, and entropy in a model which correlated significantly with intraOP pain, when excluding premenopausal women (R^2^ = 0.652) [52]. In the other study, with a similar population and anesthetic method, pain could be predicted by a mathematical model based on LF and HF, with an almost perfect ROC-curve (AUC = 0.97) [53]. From these preliminary results, preOP LF and HF could potentially be used to predict intraOP pain.

#### Postoperative pain

In carpal tunnel surgery, Nielsen et al. [54] described lower HF and rMSSD one week before surgery to be associated with increased reports of early postOP pain.

### Postoperative cardiovascular morbidity

#### Cardiac ischemia

Mamode et al. [55] found reduced triangular index 24 h before surgery in patients with either postOP cardiac death or AMI but did not distinguish between these in their analysis. In abdominal and vascular surgery, Hanss et al. found higher rates of postOP myocardial ischemia on ECG and higher total creatine kinase and creatine kinase myocardial band (CK-MB) in patients with lower TP (p < 0.05) and higher LF on the day of surgery. Other frequency domain parameters were not associated with ischemia [56]. Similarly, May et al. found that patients with HRV in the two upper tertiles of LF, HF, and rMSSD before arriving in the OR on the day of surgery had higher troponin T within the first postOP 48 h [57]. In hip fracture patients receiving arthroplasty, Laitio et al. [58] found that lower nighttime DFA α1, but not time and frequency domain analysis, predicted prolonged postOP signs of ischemia on Holter recordings in the first three postOP days. Finally, two smaller studies found no relation between HRV on the day before surgery and cardiac ischemia after non-cardiac surgery when using approximate entropy [59] or time domain measures and LF/HF [60].

In summary, there is no agreement between the studies on the predictive value of different HRV indices on the occurrence of postOP signs of cardiac ischemia.

#### Postoperative atrial fibrillation

The relation between postoperative atrial fibrillation and preOP HRV has primarily been studied on patients undergoing CABG. Studies are summarized in Table 3. Kinoshita et al. found that lower SDNN and rMSSD 1 to 5 days preOP were predictive of a lower incidence of PoAF (p < 0.01 for SDNN < 99 ms, p < 0.01 for rMSSD < 20 ms) [61]. This was not seen in two previous, smaller studies [62, 63]. Ciszewski et al. measured multiple time, frequency, and nonlinear parameters and found that lower HRV fluctuations on the day before pulmonary resection, higher rMSSD (p = 0.037), and SD1 (p = 0.036) were predictive of PoAF [64]. In cardiac surgery, Kališnik et al. found lower DFA α2 (p = 0.031) and higher pNN50 in the PoAF group (p = 0.015) [65], and Vesela et al. found lower SD2 (p = 0.009) and LF (p = 0.022) in the group experiencing PoAF, both studies measured HRV on the day before surgery [66]. In a CABG study, Chamchad et al. found that normalized preOP HF (p = 0.0302) as well as peak (p = 0.0141) and mean point correlation dimension (p = 0.033) were higher in the group with PoAF [67]. However, in a later CABG study, only lower LF/HF predicted PoAF (p = 0.0485) [68]. Both studies investigated the same time, frequency, and nonlinear measures before arriving in the OR on the day of surgery. Tarkiainen et al. found higher entropy in the PoAF group, but only analyzing nonlinear measures (p = 0.012) [69]. Bari et al. did not find a correlation between PoAF and HRV when looking at sample entropy and HF on the day of surgery [70]. Bauernschmitt et al. analyzed standard time and frequency as well as unique nonlinear parameters (HF/TP, LF/TP, “Forbidden Words”, FwRenyi025, Wsum02, Polvar10, and Shannon) on the day before surgery and found lower LF/TP, FwRenyi025, and higher “Forbidden Words” (p < 0.05 for all) to predict PoAF [14]. Three studies found lower DFA α1 on the day before surgery in groups experiencing PoAF (p = 0.015) [71] (p = 0.032) [72] (p = 0.016) [69] with Kališnik et al. also finding a trend towards higher pNN50 (p = 0.05) when analyzing multiple time, frequency and nonlinear parameters [72].

**Table 3:** Overview of studies on PoAF

Name and year	Surgery	Timing	Recording length	Measures that predicted PoAF
Wu et al. (2005) [71]	CABG (n = 86)	DBS to DOS	24 h	DFA α1
Chamchad et al. (2006) [67]	CABG (n = 88)	DOS	10 min	HF
				pPD2
				mPD2
Bauernschmitt et al. (2007) [14]	CABG or valve surgery (n = 51)	DBS	30 min	LF/TP
				FW
				FwReny025
Tarkiainen et al. (2008) [69]	CABG (n = 67)	DBS	10 min	DFA α1
Kinoshita et al. (2011) [61]	CABG (n = 390)	< 5 days before surgery	24 h	SDNN
				rMSSD
Chamchad et al. (2011) [68]	CABG (n = 50)	DOS	10 min	LF/HF
Ciszewski et al. (2013) [64]	Pulmonary resection (n = 117)	DBS	5 min	rMSSD
				SD1
Kališnik et al. (2015) [65]	CABG or valve surgery (n = 79)	DBS	20 min	pNN50
				DFA2
Vesela et al. (2019) [66]	CABG or valve surgery (n = 222)	DBS	2 h	SD1
				SD2
				rMSSD
				NN50
				LF
				HF
Kališnik et al. (2019) [72]	CABG (n = 150)	DBS	20 min	DFA α1
Jideus et al. (2001) [63]	CABG (n = 80)	DBS to DOS	24 h	No predictors
Hakala et al. (2002) [62]	CABG (n = 92)	DBS	10 min	No predictors
Bari et al. (2018) [70]	CABG (n = 129)	DOS	5 min	No predictors

*DOS* day of surgery, *DBS* day before surge

In summary, there is a large heterogeneity between methodology and timing of measurements, but reduced DFA α1 on the day before surgery might be able to predict PoAF as suggested by three of four studies.

#### Other

In pediatric neurosurgery, measures of fractal dimension by Hurst exponents obtained the day before surgery suggested that these relate to postOP hypertension while frequency analysis and approximate entropy had no predictive value [73].

### Postoperative all-cause short-term mortality (up to 30 days postOP)

Mamode et al. [55] recorded HRV 24 h before surgery and found that the preOP triangular index, but no other HRV indices, was significantly lower in patients with a compound measure of cardiac death or non-lethal AMI (p = 0.009) 30 days after peripheral arterial surgery. There was no stratification by single outcomes, limiting conclusions on the relation between HRV changes and the risk of cardiac death alone. Ernst et al. [50] measured seven HRV indices but only reported SDNN in relation to mortality, in which there was no difference betwixt patients who died and those who survived in hospital after hip arthroplasty for hip fracture. However, they did not specify the exact timing of HRV measurements in the preOP course. Filipovic et al. [74] found a higher risk of death 30 days after major non-cardiac surgery in patients with preOP LF/HF < 2.0. Zebrowski et al. [75] did not find significant differences in LF, HF, and LF/HF between patients who died within 30 days after aortic valve surgery, and those who survived. They did however find higher rMSSD (p = 0.0054) in the group that died, along with differences in other time domain analyses (lower Guzik’s, Porta’s, and Ehler’s index and higher index D) [75]. The two studies did not specify when in the preOP course HRV measurements were taken. De Godoy et al. found that several nonlinear measures (higher DFA, DFA α2, tau and lower Lyapunov, SD1 and 2) measured 24 h before surgery were associated with increased risk of death after CABG surgery, but they did not report the time of follow-up [76].

In conclusion, the predictive value of preOP HRV on postOP mortality is unclear due to heterogeneity in the available studies.

### Postoperative length of stay

#### In hospital

Two studies in abdominal and vascular surgery found that groups with lower TP had longer LOS in hospital. One measured on the day of surgery (p < 0.05) [56] and the other, one day before (p < 0.0001) [16]. However, differences in LOS were related to increased overall complications in one study [16], while causes were not reported in the other [56].

#### In intensive care unit

Two studies examined LOS in intensive care unit (ICU-LOS). One found that a group with TP < 200ms^2^ had longer ICU-LOS (p < 0.0001) following major abdominal surgery [16], and the other found lower preOP DFA α1 measured on the day before surgery predicted ICU-LOS > 24 h (p = 0.004) after CABG [71].

In conclusion, a lower preOP TP was associated with prolonged LOS both in hospital, in two studies, and in ICU in a single study.

### Other postoperative outcomes

Scheffler et al. [77] did not find a correlation between preOP HRV (time and frequency domain analysis) and postOP complications (leaks, infections, and thrombosis) after abdominal surgery. Strous et al. did not find a correlation between HRV (SDNN and rMSSD) and occurrence or severity of postOP complications in colorectal cancer surgery [78]. Ernst et al. [50] found that lower rMSSD (p < 0.05) predicted occurrence of overall complications (death, pneumonia, urinary tract infection, myocardial infarction, and stroke) and lower VLF predicted infections (p < 0.05) in hip fracture patients. In a table, they also report higher TP (p < 0.05) to predict overall complications, but in the text, they exclusively write that lower TP predicted this. The authors have been contacted regarding this apparent discrepancy, but no comment could be reached at time of publishing. We also redid the statistical analysis regarding TP and complications, as the data is publicly available, finding that higher TP was associated with complications. None of the above-mentioned studies specified when they measured HRV. Ushiyama et al. [79] separated patients in abdominal surgery depending on whether they had an uncomplicated or complicated postOP course (anastomotic leakage, delayed wound healing, or infection) but found no significant difference in SDNN or triangular index between groups when measured the day before surgery. Interestingly, they found significant pre- to postOP differences between groups (lower SDNN and triangular index). Bari et al. found significantly lower TP on the day of CABG in a group that developed acute kidney insufficiency (increase in serum creatinine during the first postOP 24 h from preOP values) compared to those that did not (p < 0.05)) [80]. Finally, a study found that nonlinear measurements on the day before surgery (DFA α1 and 2, SD1 and 2, Tau and Lyapunov) were associated with a compound measure of total complications including death [76]. Again, the data are too heterogenous to achieve any final conclusions.

## Discussion

This review on HRV and perioperative outcome in surgical patients showed that the used HRV parameters, timing of measurements, and the definitions of outcomes are too heterogeneous to support a conventional meta-analysis. However, patterns are present in the data, that allow tentative hypotheses, although all conclusions should be regarded with caution mainly due to the small size of all the included studies, which opens them up to both type I and II errors.

The primary finding for a positive association between HRV and outcome was that a high LF/HF seems to predict hypotension after SA when HRV was measured on the day of surgery. Averaging weighted means from the C-section studies that presented data suitable for this, suggests a potential cut-off value of LF/HF > 2.3 to predict occurrence of hypotension [17, 22, 27, 68].

Low frequency variability in heart rate is dominated by baroreceptor regulation and reductions in central blood volume have been shown to amplify the oscillation in this frequency range [81], as well as reduce HF in a non-hypotensive hypovolemia model [82]. It is therefore likely that the predictive value of preoperative LF/HF reflects differences in the state of hydration and intravascular volume. In the study by Hanss et al. [22], patients experiencing moderate hypotension after SA had higher LF/HF, but this was attenuated after volume treatment with 500 ml colloid. The group experiencing severe hypotension also had higher LF/HF, but this was not attenuated by volume administration. It is suggested that measurement of HRV before and after volume resuscitation can identify a subset of patients with a relative hypovolemia and therefore with an increased risk of hypotension. This subset may require more aggressive hemodynamic monitoring and intervention. However, none of the studies followed current recommendations for prophylactic vasoconstriction in C-section and the overall data on preanesthetic volume treatment and spinal hypotension is inconclusive [83].

The second finding was that low TP and a low HF seems to predict hypotension after induction of GA independent of the day of presurgical HRV measurement. In GA only two studies present data usable for such calculations, which yielded TP < 650 ms^2^ to predict occurrence of hypotension [39, 43]. TP expresses the overall HRV and longer recordings include more control systems. A reduced TP signifies a lowered autonomic regulation and hence a reduced ability to maintain homeostasis. Contrary to LF/HF, TP does not change in a model of hypotensive hypovolemia [82]. Thus, a low preoperative TP could indicate a higher, individual sensitivity to the depressant effect of anesthetics on the autonomic nervous system [84]. Adding the inhibition of autonomic control, by anesthetics, to a preexisting dysfunction is likely to cause hemodynamic maladaptation during surgery increasing the demand for vasoactive drugs [16]. However, the present data is not able to assess the specific physiological interactions between HRV indices and the cause of post-induction hypotension.

The third finding was that atrial fibrillation seems to be predicted by DFA α1 lower than 1.05 [65, 69, 71, 72]. It has been shown that DFA α1 correlates closely with LF/HF [85] and a reduction thus indicates a relative increase in parasympathetic activity to the heart. It could be that the relative increase in parasympathetic activity elicits the so-called vagal atrial fibrillation [86]. Although this is highly speculative as the physiological basis of DFA α1 is poorly understood.

The methodology used in SA studies has been relatively homogenous. Most studies measured HRV on the day of surgery, and utilized five minute recordings, with a few outliers below this recommended value [27, 36]. There were, however, large differences between studies in defining hypotension and choosing HRV indices with some studies reporting only a single index, and others reported more than five.

Contrary to those in SA, the GA studies were heterogeneous with measurements being made between several days to immediately before surgery and with ECG recording lengths varying from 30 s to 15 min. Like in SA, definitions of hypotension differed, and the number of reported indices varied from one to more than 10 in studies on GA.

In predicting postOP complications, most studies measured HRV the day before surgery, but some did not specify, and several had a range of days between measurements and surgery. In general, the studies on PostOP complication are too heterogeneous to draw any conclusions on indices, selection, or timing of measurements, except for the case of PoAF, which seems best predicted by measuring DFA α1 on the day before surgery.

Most of the reviewed studies did not attempt to correct data for preoperative comorbidities or medications, and several studies presented significant baselines differences between outcome groups. It is well known that HRV is impacted by comorbidity and cardiovascular drugs [87, 88] and hence it is not possible to determine whether HRV is a proxy for these possible confounders or if it is an independent predictor of risk. In addition, the data is sparse on the interaction of HRV with both perioperative hemodynamics, as regards both volume status and fluid management and reaction to titration of anesthetic depth and vasoactive drugs, as well as interventions to modulate the perioperative stress response.

Nonetheless, based on the review of available data, we find that a preoperative evaluation of the autonomic nervous system through HRV analysis may have a relevant role in predicting intra- and postoperative outcomes and potentially guide perioperative interventions. However, many questions remain unanswered, particularly regarding design and methodology, before recommendations and the exact utility of HRV in a surgical setting can be determined. We, therefore, propose that future studies should focus on the following: (1) Preoperative longitudinal HRV-recordings to determine the optimal time for preOP HRV assessment. (2) Outcome studies with HRV as an independent variable in a specific patient- and surgery setting with an implemented ERAS program [89]. (3) Redefining and testing cut-off HRV values prospectively. (4) Describing the interaction between perioperative hemodynamic physiology and HRV measurements. (5) Testing perioperative interventions guided by HRV indices.

This systematic review is limited by the lack of formally predefined evaluation of study quality, only using one author for screening, and the inability to perform regular meta-analysis due to the heterogeneity of the available studies.

In conclusion, we have presented an updated review of the potential use of preOP HRV in predicting perioperative complications. Predicting hypotension after spinal anesthesia in C-section and post-induction hypotension in abdominal surgery during general anesthesia seems especially promising. As does predicting postoperative atrial fibrillation. Data on other outcomes do not allow sufficient interpretation, thereby calling for better design of future studies within standardized perioperative setups [89].

## Supplementary Information

Below is the link to the electronic supplementary material.

Supplementary file1 (DOCX 325 KB)

## Data Availability

All used studies have been referenced and are published.

## Footnotes

1 Such as fast fourier transformation, autoregressive analysis and wavelet transformation.
