# Supplementary material for: Preoperative heart rate variability as a predictor of perioperative outcomes: a systematic review without meta-analysis
Source: J Clin Monit Comput. 2022 Jan 29;36(4):947–60. doi: 10.1007/s10877-022-00819-z (PMC9293802; doi:10.1007/s10877-022-00819-z)
Supplement: Supplementary file 1 — Supplementary file1 (DOCX 325 KB) [file 10877_2022_819_MOESM1_ESM.docx]

# Supplementary tables

**Article title:**

Preoperative heart rate variability as a predictor of perioperative outcomes: A systematic review without metaanalysis.

**Journal name:**

Journal of Clinical Monitoring and Computing

**Author names and affiliations:**

Mikkel Nicklas Frandsen MS.^1^ ORCiD: 0000-0001-8368-314X; Jesper Mehlsen MD^1^ ORCiD: 0000-0002-1720-0581; Nicolai Bang Foss, MD, DMMSc^2^ ORCiD: 0000-0002-9376-3845; Henrik Kehlet MD, PhD^1^ ORCiD: 0000-0002-2209-1711

^1^ Section for Surgical Pathophysiology, Copenhagen University Hospital, Rigshospitalet Denmark

^2^ Department of Anaesthesiology and Intensive care, Hvidovre University Hospital, Denmark

**Corresponding author:**

Mikkel Nicklas Frandsen MS.

Email: mikkel.nicklas.frandsen@regionh.dk

| **Pre to intraOP** | **Definition** | **Positive findings** | **Negative findings** |
| --- | --- | --- | --- |
| **Time domain frequency** |  |  |  |
| SDNN | Standard deviation of NNI | ↓ predicted hypotension(2GA + 1SA)[15,30,43] | Did not predict hypotension (SA)[29] |
| SDRR | Standard deviation of all RR intervals |  | Did not predict hypotension(SA)[33] |
| rMSSD | Square root of the mean squared differences between NNI. | ↓ predicted hypotension(2GA + 1SA)[30,43,46]  ↓ predicted positive OCR[47] | Did not predict hypotension(1GA)[44] |
| pNN50 | Percentage of NNI differing by more than 50 ms | ↓ predicted positive OCR[47] | Did not predict hypotension(1GA)[44] |
| Coefficient of variation (CV) | The SDNN divided by the mean NNI | ↓ predicted hypotension(GA)[46] |  |
| **Frequency domain** |  |  |  |
| Total power (TP)§ | The variance of the NNI | ↓predicted hypotension(5GA)[15,39,42,43,45]  ↓predicted higher blood loss[16]  ↓predicted need for vasopressors[16]  ↓predicted bradycardia[39] | Did not predict hypotension(1GA + 3SA + 1EPI)[32–34,37,41] |
| High Frequency (HF)*† | The variance of the NNI in the high frequency spectrum. Can be normalized by dividing with total power minus VLF | ↓predicted hypotension(6GA + 1SA)[15,16,33,41,43,45,46]  ↓predicted hypotension (normalized)(1SA)[22]  ↑ predicted bradycardia[38]  ↓predicted bradycardia[48]  ↓Predicted OCR[47]  ↑predicted lower intraOP pain[52,53] | Did not predict hypotension(1GA+2SA+1EPI)[17,30,37,44]  Did not predict hypotension (normalized)(1GA+1SA)[26,39] |
| Low Frequency (LF)* | The variance of the NNI in the low frequency spectrum. Can be normalized by dividing with total power minus VLF | ↓predicted hypotension(5GA+1SA)[15,16,30,43,45,46]  ↑predicted hypotension (normalized)(2SA)[22,33]  ↑predicted lower intraOP pain[52] | Did not predict hypotension(2GA+1SA+1EPI)[17,37,41,44]  Did not predict OCR[47]  Did not predict hypotension (normalized)(1GA+1SA)[26,39]  Did not predict bradycardia[38,48]  Did not predict intraOP pain[53] |
| Very low frequency (VLF)* | The variance of the NNI in the very low frequency spectrum. |  | Did not predict hypotension(2GA)[41,46] |
| LF/HF ratio† | LF divided by HF | ↑ predicted hypotension(2GA+5SA+1EPI)[22–24,31–33,37,41,43]  ↑ predicted need of vasopressors[16]  ↑ predicted bloodloss[16]  ↑ predicted bradycardia[48] | Did not predict hypotension(3GA+5SA)[17,27,30,34,35,39,44,45]  Did not predict hypotension (trend)(SA)[26]  Did not predict bradycardia[38] |
| LF permutation entropy | A measure of complexity in the LF band | ↑predicted pain[53] |  |
| HF permutation entropy | A measure of complexity in the HF band |  | Did not predict pain[53] |
| Analgesia nociception index (ANI) | A measure of respiratory sinus arrythmia, similar to HF |  | Did not predict hypotension(1SA)[28] |
| **Nonlinear methods** |  |  |  |
| Poincaré SD1 | A measure of short-term variability | ↓ predicted hypotension[43] |  |
| Poincaré SD2 | A measure of long-term variability | ↓ predicted hypotension[43] |  |
| Fractal dimension | A measure of irregularity. | ↓ predicted OCR[47] |  |
| Detrended fluctuation analysis (DFA) α1 | Short-term correlation between successive RR intervals. |  | Did not predict hypotension[43] |
| Detrended fluctuation analysis (DFA) α2 | Long-term correlation between successive RR intervals. |  | Did not predict hypotension[43] |
| Sample entropy (SampEn) | A measure of randomness in a signal. Higher values equal more randomness. |  | Did not predict hypotension[17,43] |
| Approximate entropy (ApEn) | A measure of randomness in a signal. Higher values equal more randomness. | ↓ predicted hypotension[17] | Did not predict OCR[47] |
| Ultra-short entropy (UsEn)‡ | A measure of randomness in a signal. Higher values equal more randomness. | ↓predicted hypotension [36]  ↓predicted need for ephedrine[40] | Did not predict hypotension[27] |
| Correlation dimension (D2) | Number of independent variables needed to define a system | ↓ predicted hypotension[43] | Did not predict hypotension[17] |
| Peak PD2 (pPD2) | Peak number of independent sources of information in the HRV signal | ↓ Predicted hypotension[26] |  |
| Prediction error | The ability of an algorithm to predict the second half of a dataset after training on the first half | ↓ predicted positive OCR[47] |  |
| ANSindex | No publicly available details on measurement. Reported to measure the sympathetic outflow. | ↑ increased risk of hypotension(1SA)[25] |  |

**Supplementary table 1: Preoperative HRV indices and intraoperative outcome**

*: Knüttgen and Latson et al does not define HF and LF like other studies.
^†:^ Bolea utilizes a variable HF, therefore results will not be entirely comparable to others.
‡: Yokose does not define entropy, but we guess that it is UsEn based on number of RR-intervals analyzed

| **Pre to PostOP** | **What is it** | **Positive findings** | **Negative findings** |
| --- | --- | --- | --- |
| **Time domain frequency** |  |  |  |
| SDNN | Standard deviation of normal-to-normal intervals | ↑predicted PoAF[61] | Did not predict death[50,75]  Did not predict AMI or cardiac death (aggregate measure)[55]  Did not predict infections[50,79]  Did not predict pneumonia[50]  Did not predict UTI[50]  Did not predict anastomotic leaks[79]  Did not predict delayed wound healing[79]  Did not predict postoperative pain[54]  Did not predict prolonged myocardial ischaemia[57,58]  Did not predict PoAF[14,62–65,67,68,70,72]  Did not predict overall complications[50,78] |
| SDRR | Standard deviation of R-peak to R-peak interval (unadjusted for non-sinus rhythm R-peaks) |  | Did not predict ischaemia[60] |
| SDANN | Standard deviation of NNI, averaged over 5-minute segments |  | Did not predict ischaemia[60]  Did not predict postOP death[75]  Did not predict AMI or cardiac death(aggregate measure)[55]  Did not predict PoAF[63] |
| rMSSD | Square root of the mean squared differences between normal-to normal-intervals | ↓predicted overall complications[50]  ↑predicted death[75]  ↑predicted AF[61,64]  Difference predicted AF (unspecified)[66] | Did not predict pain[54]  Did not predict myocardial ischaemia[57]  Did not predict AF[14,62,63,65,67,68,72]  Did not predict overall complications[78]  Did not predict infections[50]  Did not predict pneumonia[50]  Did not predict UTI[50] |
| NN50 | Number of NNI differing by more than 50ms | Difference predicted AF (unspecified)[66] | Did not predict AF[64,68] |
| pNN50 | Percentage of normal-normal intervals differing by more than 50 ms | ↑predicted AF[65,72] | Did not predict AMI or cardiac death (aggregate measure)[55]  Did not predict AF[64,67,68]  Did not predict anastomotic leaks[77]  Did not predict infection[77] |
| Triangular index | Total NNI intervals divided by the categorical mode of NNI | ↓ predicted AMI or cardiac death[55] | Did not predict infection[77,79]  Did not predict anastomotic leaks[77,79]  Did not predict delayed wound healing[79]  Did not predict AF[67,68] |
| TINN | Width of the baseline RR histogram used in triangular index analysis |  | Did not predict AF[67,68] |
| Guzik’s index (G%) | Ratio of the sum of the squares of the positive consecutive differences of RR intervals to the sum of the squares  of all consecutive RR interval differences | ↓predicted postOP death[75] |  |
| Porta’s index (P%) | the ratio of the number of  negative consecutive differences of RR intervals to the total number of non-zero differences of RR intervals | ↓ predicted postOP death[75] |  |
| Index D (D) | The square root of the sum of Porta’s and Guzik’s indexes subtracted 50 then squared | ↑ predicted postOP death[75] |  |
| Ehler’s index (E) | The skewness of the probability distribution  of the RR intervals | ↓ predicted postOP death[75] |  |
| **Frequency domain** |  |  |  |
| Total power (TP) | The variance of the NNI | ↓predicted overall complications[16]  ↑ predicted overall complications[50]  ↓predicted LOS[16,56]  ↓predicted ICU-LOS[16]  ↓ predicted signs of myocardial ischaemia[56]  ↓ predicted AKD[80] | Did not predict pain[54]  Did not predict AF[62,63,65,72]  Did not predict myocardial ischaemia[60]  Did not predict postOP hypertension[73]  Did not predict infections[50]  Did not predict pneumonia[50]  Did not predict UTI[50] |
| High Frequency (HF) | The variance of the NNI in the high frequency spectrum. Can be normalized by dividing with total power minus VLF | ↓predicted overall complications[16]  ↓Predicted early postOP pain[54]  ↑ predicted AF[67]  Difference predicted AF (unspecified)[66] | Did not predict overall complications[50,77]  Did not predict death[75]  Did not predict AF[14,62–65,68,70,72]  Did not predict ischaemeia[57,58]  Did not predict postOP hypertension[73]  Did not predict adverse respiratory events when emerging from anesthesia[49]  Did not predict infections[50]  Did not predict pneumonia[50]  Did not predict UTI[50] |
| Coefficient component variance-HF (CCV-HF) | HF adjusted for difference in heart rate | ↓ predicted postOP pain[54] |  |
| Low Frequency (LF) | The variance of the NNI in the low frequency spectrum. Can be normalized by dividing with total power minus VLF | ↓predicted overall complications[16]  ↑predicted ischaemia[57]  ↓predicted AF(normalized LF)[14]  ↓predicted AF[66] | Did not predict overall complications[50,77]  Did not predict postOP death[75]  Did not predict pain[54]  Did not predict myocardial ischaemia[58]  Did not predict AF[14,62–65,67,68,72]  Did not predict respiratory adverse events after anesthesia[49]  Did not predict infections[50]  Did not predict pneumonia[50]  Did not predict UTI[50] |
| Coefficient component variance-LF (CCV-LF) | LF adjusted for difference in heart rate | ↓ predicted postOP pain[54] |  |
| Very low frequency (VLF) | The variance of the NNI in the very low frequency spectrum. | ↓predicted infections in general[50]  ↓predicted UTI[50] | Did not predict postOP ischaemia[58]  Did not predict AF[62–65,72]  Did not predict respiratory adverse events after anesthesia[49]  Did not predict overall complications[50]  Did not predict pneumonia[50] |
| LF/HF ratio | LF divided by HF | ↑predicted overall complications[16]  ↓predicted pneumonia[50]  ↓Predicted AF[68]  ↓predicted postOP death[74] | Did not predict postOP death[75]  Did not predict AF (trend)[67]  Did not predict AF[62–65,72]  Did not predict overall complications[50,77]  Did not predict postOP hypertension[73]  Did not predict respiratory adverse events after anesthesia[49]  Did not predict postOP ischaemia[56,60]  Did not predict infections[50]  Did not predict UTI[50] |
| **Nonlinear methods** |  |  |  |
| Symbolic dynamic entropy (also called Shannon) | RR intervals are transformed to numbers 0-3 depending on difference from mean, and entropy analysis is then performed on the data. | ↓ predicted AF[69] | Did not predict PoAF[14] |
| Forbidden words (FW) | A measure of symbolic dynamics. Higher values indicate lower HRV. | ↑ predicted PoAF[14] |  |
| FwRenyi025 | A measure of symbolic dynamics. Higher values indicate higher complexity. | ↓ predicted PoAF[14] |  |
| Wpsum02 | A measure of symbolic dynamics. Higher values indicate lower HRV. |  | Did not predict PoAF[14] |
| Polvar10 | A measure of symbolic dynamics. The probability of 6 successive beats differing by less than 10ms from the mean. |  | Did not predict PoAF[14] |
| Tau | A measure of correlation between NNI and the previous NNI. Measures linearity. | ↓predicted overall complications and death (aggregate measure)[76]  ↓predicted death[76] |  |
| Poincaré SD1 | A measure of short-term variability | ↑predicted AF[64]  Predicted AF (unspecified)[66]  ↓predicted overall complications and death (aggregate measure)[76]  ↓predicted death[76] | Did not predict AF[67,68] |
| Poincaré SD2 | A measure of long-term variability | ↓Predicted AF[66]  ↓Predicted overall complications and death (aggregate measure)[76]  ↓predicted death[76] | Did not predict AF[64,67,68] |
| SD2/SD1 | SD2 divided by SD1 |  | Did not predict AF[64] |
| Lyapunov exponent | A measure of chaos. Higher values indicate more chaos and less predictability. | ↓Predicted overall complications and death (aggregate measure)[76]  ↓predicted death[76]  ↓Predicted pulmonary infection[51] |  |
| Hurst exponent | A measure of long-term correlation | ↑Predicted death[75]  ↑Predicted postOP hypertension[73] | Did not predict pulmonary infection[51] |
| Fractal Dimension | A measure of irregularity. |  | Did not predict AF (trend)[65] |
| Total detrended fluctuation analysis (DFA) | Correlation between successive RR intervals | ↑predicted pulmonary infection[51]  ↑Predicted death[76] | Did not predict overall complications and death (aggregate measure)[76] |
| DFA α1 | Short-term correlation between successive RR intervals. | ↓ at night predicted myocardial ischaemia[58]  ↓ predicted AF[69,71,72]  ↓predicted overall complications and death (aggregate measure)[76]  ↓predicted longer ICU-LOS[71]  ↓predicted need for inotropes[71] | Did not predict AF[65]  Did not predict death[76]  Did not predict pulmonary infections[51]  Did not predict respirator use[71] |
| DFA α2 | Long-term correlation between successive RR intervals. | ↓ predicted AF[65]  ↓ predicted overall complications and death (aggregate measure)[76]  ↓ predicted death[76] | Did not predict AF[72]  Did not predict pulmonary infections (trend)[51] |
| Sample entropy (SampEn) | A measure of randomness in a signal. Higher values equal more randomness. |  | Did not predict AF[70] |
| Approximate entropy (ApEn) | A measure of randomness in a signal. Higher values equal more randomness. | ↓predicted pulmonary infection[51] | Did not predict postOP hypertension[73]  Did not predict AF[69]  Did not predict ventricular dysfunction[59] |
| Embedded spectral entropy | A measure of complexity |  | Did not predict AF[68] |
| pPD2 | Peak number of independent sources of information in the HRV signal | ↑ predicted AF[67] | Did not predict AF[68] |
| mPD2 | Mean number of independent sources of information in the HRV signal | ↑ predicted AF[67] | Did not predict AF[68] |

**Supplementary table 2: Preoperative HRV indices and postoperative outcome**

| **Pre to intraOP** | **Predicted by** | **Not predicted by** |
| --- | --- | --- |
| **Complication** |  |  |
| Hypotension | ↓SDNN(2GA + 1SA)[15,30,43]  ↓ rMSSD(2GA + 1SA)[30,43,46]  ↓ CV (GA)[46]  ↑ ANSindex (1SA)[25]  ↓TP(5GA)[15,39,42,43,45]  ↓HF(6GA + 1SA)[15,16,33,41,43,45,46]  ↓HF (normalized) predicted hypotension (1SA)[22]  ↓LF(5GA+1SA)[15,16,30,43,45,46]  ↑LF(normalized)(2SA)[22,33]  ↑LF/HF(2GA+6SA+1EPI)[22–24,31–33,37,41,43]  ↓Poincaré SD1[43]  ↓Poincaré SD2[43]  ↓ ApEn[17]  ↓UsEn[36]  ↓ D2[43]  ↓ pPD2[26] | SDNN(SA)[29]  SDRR (SA)[33]  pNN50 (1GA)[44]  TP (1GA + 3SA + 1EPI)[32–34,37,41]  HF (1GA+2SA+1EPI)[17,30,37,44]  HF (normalized) (1GA+1SA)[26,39]  LF (2GA+1SA+1EPI)[17,37,41,44]  LF (normalized) (1GA+1SA)[26,39]  VLF (2GA)[41,46]  LF/HF (3GA+5SA)[17,27,30,34,35,39,44,45]  LF/HF (trend)(SA)[26]  ANI (1SA)[28]  DFA α1[43]  DFA α2[43]  SampEn[17,43]  UsEn[27]  D2[17] |
| Oculocardiac reflex | ↓ rMSSD[47]  ↓ pNN50[47]  ↓ HF[47]  ↓ Fractal dimension[47]  ↓ Prediction error[47] | LF[47]  ApEn[47] |
| Need for vasopressors | ↓ TP[16]  ↑ LF/HF[16,31]  ↓UsEn[40] |  |
| Blood loss | ↓ TP[16]  ↑ LF/HF[16] |  |
| Bradycardia | ↓TP predicted bradycardia[39]  ↑ HF predicted bradycardia[38]  ↓HF predicted bradycardia[48]  ↑ LF/HF predicted bradycardia[48] | LF[38,48]  LF/HF[38] |
| intraOP pain | ↑HF predicted lower pain[52,53]  ↑LF predicted lower pain[52]  ↑LFPE predicted higher pain[53] | LF[53]  HFPEn[53] |

**Supplementary table 3: Intraoperative complications and HRV indices in relation to them.**

| **Pre to postOP** | **Predicted by** | **Not predicted by** |
| --- | --- | --- |
| **Complication** |  |  |
| All-cause mortality | ↑rMSSD[75]  ↓ triangular index[55]  ↓Guzik’s index[75]  ↓Porta’sindex[75]  ↓Ehlers index[75]  ↑Index D[75]  ↓LF/HF[74]  ↓ Tau[76]  ↓ SD1[76]  ↓ SD2[76]  ↓ DFA[76]  ↓ DFA α2[76]  ↓ Lyapunov[76]  ↑ Hurst[75] | SDNN[50,75]  SDANN[75]  pNN50[55]  HF[75]  HF[75]  LF/HF[75]  DFA α1[76] |
| Cardiac event (AMI or cardiac death | ↓ Triangular index[55] | SDNN[55]  SDANN[55]  pNN50[55] |
| Signs of ischaemia | ↓Triangular index[55]  ↓TP[56]  ↑LF[56]  ↓ DFA α1 at night[58] | SDNN[55,57,58]  SDANN[55,60]  SDRR[60]  rMSSD[57]  pNN50[55]  TP[60]  HF[56–58]  LF[57,58]  LF/HF[56,60]  VLF[58]  ApEn[59] |
| Need for inotropes/vasopressors | ↓ DFA α1[71] |  |
| Ventricular dysfunction |  | ApEn[59] |
| Postoperative atrial fibrillation | ↑SDNN[61]  ↑rMSSD[61,64]  rMSSD (unspecified difference)[66]  NN50 (unspecified difference)[66]  ↑pNN50[65,72]  ↑HF[67]  HF (unspecified difference)[66]  ↓LF[66]  ↓LF (normalized)[14]  ↓LF/HF[68]  ↓SymDynEn[69]  ↑FW[14]  ↓FWRenyi025[14]  ↑SD1[64]  SD1 (unspecified difference) [66]  ↓SD2[66]  ↓DFA α1[69,71,72]  ↓DFA α2[65]  ↑ pPD2[67]  ↑ mPD2[67] | SDNN[14,62–65,67,68,70,72]  SDANN[63]  rMSSD[14,62,63,65,67,68,72]  NN50[64,68]  pNN50[64,67,68]  Triangular index[67,68]  TINN[67,68]  TP[62,63,65,72]  HF[14,62–65,68,70,72]  LF[14,62–65,67,68,72]  VLF[62–65,72]  LF/HF[62–65,67,72]  SymDynEn[14]  Wpsum02[14]  Polvar10[14]  SD1[67,68]  SD2[64,67,68]  SD2/SD1[64]  FD[65]  DFA α1[65]  DFA α2[72]  SampEn[70]  ApEn[69]  pPD2[68]  mPD2[68] |
| Hypertension | ↑Hurst[73] | TP[73]  HF[73]  LF/HF[73]  ApEn[73] |
| Respiratory events after anesthesia |  | HF[49]  LF[49]  VLF[49]  LF/HF[49] |
| Respirator use |  | DFA1[71] |
| Overall complications (several aggregate measures) | ↓rMSSD[50]  ↓TP[16]  ↑TP[50]  ↓HF[16]  ↓LF[16]  ↑LF/HF[16]  ↓Tau[76]  ↓SD1[76]  ↓SD2[76]  ↓Lyapunov[76]  ↓DFA α1[76]  ↓DFA α2[76] | SDNN[50,78,79]  rMSSD[78]  pNN50[77]  Triangular index[77,79]  HF[50,77]  LF[50,77]  VLF[50]  LF/HF[50,77]  DFA[76] |
| Infections in general | ↓VLF[50] | SDNN[50]  rMSSD[50]  TP[50]  HF[50]  LF[50]  LF/HF[50] |
| UTI | ↓VLF[50] | SDNN[50]  rMSSD[50]  TP[50]  HF[50]  LF[50]  LF/HF[50] |
| Pneumonia/pulmonary infection | ↓LF/HF[50]  ↑DFA[51]  ↓ApEn[51]  ↓Lyapunov[51] | SDNN[50]  rMSSD[50]  TP[50]  HF[50]  LF[50]  VLF[50]  DFA α1[51]  DFA α2[51]  Hurst[51] |
| AKD | ↓TP[80] |  |
| Pain | ↓HF[54]  ↓CCV-HF[54]  ↓CCV-LF[54] | SDNN[54]  rMSSD[54]  TP[54]  LF[54] |
| LOS | ↓TP[16,56] |  |
| ICU-LOS | ↓TP[16]  ↓DFA1[71] |  |

**Supplementary table 4:** Postoperative complications and HRV indices in relation to them.

# References

1. Shaffer F, Ginsberg JP. An Overview of Heart Rate Variability Metrics and Norms. Front Public Health [Internet]. 2017 [cited 2020 Dec 14];5. https://doi.org/10.3389/fpubh.2017.00258

2. Kamath MV, Watanabe M, Upton A. Heart Rate Variability (HRV) Signal Analysis: Clinical Applications. CRC Press; 2012.

3. Wolf MM, Varigos GA, Hunt D, Sloman JG. Sinus arrhythmia in acute myocardial infarction. Med J Aust. 1978;2:52–3. https://doi.org/10.5694/j.1326-5377.1978.tb131339.x

4. Kleiger RE, Miller JP, Bigger JT, Moss AJ. Decreased heart rate variability and its association with increased mortality after acute myocardial infarction. Am J Cardiol. 1987;59:256–62. https://doi.org/10.1016/0002-9149(87)90795-8

5. Mäkikallio TH, Barthel P, Schneider R, Bauer A, Tapanainen JM, Tulppo MP, et al. Prediction of sudden cardiac death after acute myocardial infarction: role of Holter monitoring in the modern treatment era. Eur Heart J. 2005;26:762–9. https://doi.org/10.1093/eurheartj/ehi188

6. Kawamoto M, Tanaka N, Takasaki M. Power spectral analysis of heart rate variability after spinal anaesthesia. Br J Anaesth. 1993;71:523–7. https://doi.org/10.1093/bja/71.4.523

7. Laitio T, Jalonen J, Kuusela T, Scheinin H. The role of heart rate variability in risk stratification for adverse postoperative cardiac events. Anesth Analg. 2007;105:1548–60. https://doi.org/10.1213/01.ane.0000287654.49358.3a

8. Electrophysiology Task Force of the European Society of Cardiology the North American Society of Pacing. Heart Rate Variability. Circulation. American Heart Association; 1996;93:1043–65. https://doi.org/10.1161/01.CIR.93.5.1043

9. Henriques T, Ribeiro M, Teixeira A, Castro L, Antunes L, Costa-Santos C. Nonlinear Methods Most Applied to Heart-Rate Time Series: A Review. Entropy. Multidisciplinary Digital Publishing Institute; 2020;22:309. https://doi.org/10.3390/e22030309

10. Logier R, Jeanne M, De Jonckheere J, Dassonneville A, Delecroix M, Tavernier B. PhysioDoloris: a monitoring device for analgesia / nociception balance evaluation using heart rate variability analysis. Annu Int Conf IEEE Eng Med Biol Soc IEEE Eng Med Biol Soc Annu Int Conf. United States; 2010;2010:1194–7. https://doi.org/10.1109/IEMBS.2010.5625971

11. Goldstein DS, Bentho O, Park M-Y, Sharabi Y. Low-frequency power of heart rate variability is not a measure of cardiac sympathetic tone but may be a measure of modulation of cardiac autonomic outflows by baroreflexes. Exp Physiol. 2011;96:1255–61. https://doi.org/https://doi.org/10.1113/expphysiol.2010.056259

12. Godoy MF de. Nonlinear Analysis of Heart Rate Variability: A Comprehensive Review. J Cardiol Ther. 2016;3:528–33.

13. Lafitte MJ, Sauvageot OR, Fevre-Genoulaz M, Zimmermann M. Towards assessing the sympathovagal balance. Med Biol Eng Comput. 2006;44:675–82. https://doi.org/10.1007/s11517-006-0053-1

14. Bauernschmitt R, Malberg H, Wessel N, Brockmann G, Wildhirt SM, Kopp B, et al. Autonomic control in patients experiencing atrial fibrillation after cardiac surgery. Pacing Clin Electrophysiol PACE. United States; 2007;30:77–84. https://doi.org/10.1111/j.1540-8159.2007.00568.x

15. Latson TW, Ashmore TH, Reinhart DJ, Klein KW, Giesecke AH. Autonomic Reflex Dysfunction in Patients Presenting for Elective Surgery Is Associated with Hypotension after Anesthesia Induction. Anesthesiology. American Society of Anesthesiologists; 1994;80:326–37. https://doi.org/10.1097/00000542-199402000-00013

16. Reimer P, Máca J, Szturz P, Jor O, Kula R, Ševčík P, et al. Role of heart-rate variability in preoperative assessment of physiological reserves in patients undergoing major abdominal surgery. Ther Clin Risk Manag. 2017;13:1223–31. https://doi.org/10.2147/TCRM.S143809

17. Bolea J, Lázaro J, Gil E, Rovira E, Remartínez JM, Laguna P, et al. Pulse Rate and Transit Time Analysis to Predict Hypotension Events After Spinal Anesthesia During Programmed Cesarean Labor. Ann Biomed Eng. 2017;45:2253–63. https://doi.org/10.1007/s10439-017-1864-y

18. Ackland GL, Abbott TEF, Minto G, Clark M, Owen T, Prabhu P, et al. Heart rate recovery and morbidity after noncardiac surgery: Planned secondary analysis of two prospective, multi-centre, blinded observational studies. PLOS ONE. Public Library of Science; 2019;14:e0221277. https://doi.org/10.1371/journal.pone.0221277

19. Abbott TEF, Pearse RM, Cuthbertson BH, Wijeysundera DN, Ackland GL, METS study investigators. Cardiac vagal dysfunction and myocardial injury after non-cardiac surgery: a planned secondary analysis of the measurement of Exercise Tolerance before surgery study. Br J Anaesth. 2019;122:188–97. https://doi.org/10.1016/j.bja.2018.10.060

20. Schardt C, Adams MB, Owens T, Keitz S, Fontelo P. Utilization of the PICO framework to improve searching PubMed for clinical questions. BMC Med Inform Decis Mak. 2007;7:16. https://doi.org/10.1186/1472-6947-7-16

21. Liberati A, Altman DG, Tetzlaff J, Mulrow C, Gøtzsche PC, Ioannidis JPA, et al. The PRISMA Statement for Reporting Systematic Reviews and Meta-Analyses of Studies That Evaluate Health Care Interventions: Explanation and Elaboration. PLoS Med [Internet]. 2009 [cited 2020 Nov 3];6. https://doi.org/10.1371/journal.pmed.1000100

22. Hanss R, Bein B, Ledowski T, Lehmkuhl M, Ohnesorge H, Scherkl W, et al. Heart rate variability predicts severe hypotension after spinal anesthesia for elective cesarean delivery. Anesthesiology. 2005;102:1086–93. https://doi.org/10.1097/00000542-200506000-00005

23. Hanss R, Bein B, Francksen H, Scherkl W, Bauer M, Doerges V, et al. Heart rate variability-guided prophylactic treatment of severe hypotension after subarachnoid block for elective cesarean delivery. Anesthesiology. 2006;104:635–43. https://doi.org/10.1097/00000542-200604000-00005

24. Bishop DG, Cairns C, Grobbelaar M, Rodseth RN. Heart rate variability as a predictor of hypotension following spinal for elective caesarean section: a prospective observational study. Anaesthesia. 2017;72:603–8. https://doi.org/10.1111/anae.13813

25. Prashanth A, Chakravarthy M, George A, Mayur R, Hosur R, Pargaonkar S. Sympatho-vagal balance, as quantified by ANSindex, predicts post spinal hypotension and vasopressor requirement in parturients undergoing lower segmental cesarean section: a single blinded prospective observational study. J Clin Monit Comput. 2017;31:805–11. https://doi.org/10.1007/s10877-016-9906-9

26. Chamchad D, Arkoosh VA, Horrow JC, Buxbaum JL, Izrailtyan I, Nakhamchik L, et al. Using heart rate variability to stratify risk of obstetric patients undergoing spinal anesthesia. Anesth Analg. 2004;99:1818–21. https://doi.org/10.1213/01.ANE.0000140953.40059.E6

27. Yokose M, Mihara T, Sugawara Y, Goto T. The predictive ability of non-invasive haemodynamic parameters for hypotension during caesarean section: a prospective observational study. Anaesthesia. 2015;70:555–62. https://doi.org/10.1111/anae.12992

28. Jendoubi A, Khalloufi A, Nasri O, Abbes A, Ghedira S, Houissa M. Analgesia nociception index as a tool to predict hypotension after spinal anaesthesia for elective caesarean section. J Obstet Gynaecol J Inst Obstet Gynaecol. 2020;1–7. https://doi.org/10.1080/01443615.2020.1718624

29. Helmy Shehata J., Ibrahim El Sakka A., Omran A., Abdeltawab Mahmoud Atia Gbre M., Mohamed A.A., Fetouh A.M., et al. Heart rate variability as a predictor of hypotension following spinal anesthesia for elective caesarian section in preeclamptic parturients: A descriptive observational study. Open Access Maced J Med Sci. North Macedonia: Open Access Macedonian Journal of Medical Sciences (E-mail: mspiroski@id-press.eu); 2019;7:4043–7. https://doi.org/10.3889/oamjms.2019.703

30. Vinayagam S, Panta S, Badhe A, Sharma V. Heart rate variability as a predictor of hypotension after spinal anaesthesia in patients with diabetes mellitus. Indian J Anaesth. 2019;63:671. https://doi.org/10.4103/ija.IJA_13_19

31. Sharma D, Gupta K, Gupta P, Tyagi SK. Heart rate variability assessment to stratify risk of autonomic imbalance during subarachnoid block: A prospective study. Anesth Essays Res. 2011;5:72–6. https://doi.org/10.4103/0259-1162.84200

32. Raimondi F, Colombo R, Spazzolini A, Corona A, Castelli A, Rech R, et al. Preoperative autonomic nervous system analysis may stratify the risk of hypotension after spinal anesthesia. Minerva Anestesiol. 2015;81:713–22.

33. Hanss R, Bein B, Weseloh H, Bauer M, Cavus E, Steinfath M, et al. Heart rate variability predicts severe hypotension after spinal anesthesia. Anesthesiology. 2006;104:537–45. https://doi.org/10.1097/00000542-200603000-00022

34. Kweon TD, Kim SY, Cho SA, Kim JH, Kang YR, Shin Y-S. Heart rate variability as a predictor of hypotension after spinal anesthesia in hypertensive patients. Korean J Anesthesiol. The Korean Society of Anesthesiologists; 2013;65:317–21. https://doi.org/10.4097/kjae.2013.65.4.317

35. Meyhoff CS, Haarmark C, Kanters JK, Rasmussen LS. Is it possible to predict hypotension during onset of spinal anesthesia in elderly patients? J Clin Anesth. 2009;21:23–9. https://doi.org/10.1016/j.jclinane.2008.06.015

36. Fujiwara Y, Sato Y, Shibata Y, Asakura Y, Nishiwaki K, Komatsu T. A greater decrease in blood pressure after spinal anaesthesia in patients with low entropy of the RR interval. Acta Anaesthesiol Scand. 2007;51:1161–5. https://doi.org/10.1111/j.1399-6576.2007.01435.x

37. Owczuk R, Steffek M, Wujtewicz M, Marjanski T, Wujtewicz M. Heart rate variability may predict high risk of hypotension due to thoracic epidural anaesthesia. Eur J Anaesthesiol. R. Owczuk, Department of Anaesthesiology and Intensive Therapy, Medical University of Gdansk, Gdansk, Poland: Lippincott Williams and Wilkins; 2009;26:113.

38. Chatzimichali A, Zoumprouli A, Metaxari M, Apostolakis I, Daras T, Tzanakis N, et al. Heart rate variability may identify patients who will develop severe bradycardia during spinal anaesthesia. Acta Anaesthesiol Scand. 2011;55:234–41. https://doi.org/10.1111/j.1399-6576.2010.02339.x

39. Hanss R, Renner J, Ilies C, Moikow L, Buell O, Steinfath M, et al. Does heart rate variability predict hypotension and bradycardia after induction of general anaesthesia in high risk cardiovascular patients? Anaesthesia. 2008;63:129–35. https://doi.org/10.1111/j.1365-2044.2007.05321.x

40. Fujiwara Y, Ito H, Asakura Y, Sato Y, Nishiwaki K, Komatsu T. Preoperative ultra short-term entropy predicts arterial blood pressure fluctuation during the induction of anesthesia. Anesth Analg. 2007;104:853–6. https://doi.org/10.1213/01.ane.0000258756.41649.2d

41. Raghavan LV. Heart rate variability predicts post-induction hypotension in patients with cervical myelopathy. Can J Anesth. L.V. Raghavan, Toronto Western Hospital, University of Toronto: Springer; 2019;66:S373–S374. https://doi.org/http://dx.doi.org/10.1007/s12630-019-01499-1

42. Dinesh N, Geetha SM. Pre-operative screening of diabetic patients for heart rate variability and their hemodynamic responses during induction of general anaesthesia. Indian J Anaesth. N. Dinesh, M.S.Ramaiah Medical College, Bangalore, India: Wolters Kluwer Medknow Publications; 2020;64:S4–S5. https://doi.org/http://dx.doi.org/10.4103/0019-5049.277899

43. Padley JR, Ben-Menachem E. Low pre-operative heart rate variability and complexity are associated with hypotension after anesthesia induction in major abdominal surgery. J Clin Monit Comput. 2018;32:245–52. https://doi.org/10.1007/s10877-017-0012-4

44. Huh IY, Kim D-Y, Lee J-H, Shin SJ, Cho YW, Park SE. Relation between preoperative autonomic function and blood pressure change after tourniquet deflation during total knee replacement arthroplasty. Korean J Anesthesiol. 2012;62:154–60. https://doi.org/10.4097/kjae.2012.62.2.154

45. Huang C-J, Kuok C-H, Kuo TBJ, Hsu Y-W, Tsai P-S. Pre-operative measurement of heart rate variability predicts hypotension during general anesthesia. Acta Anaesthesiol Scand. 2006;50:542–8. https://doi.org/10.1111/j.1399-6576.2006.001016.x

46. Knüttgen D, Trojan S, Weber M, Wolf M, Wappler F. [Pre-operative measurement of heart rate variability in diabetics: a method to estimate blood pressure stability during anaesthesia induction]. Anaesthesist. 2005;54:442–9. https://doi.org/10.1007/s00101-005-0837-y

47. Kim HS, Kim SD, Kim CS, Yum MK. Prediction of the oculocardiac reflex from pre-operative linear and nonlinear heart rate dynamics in children. Anaesthesia. 2000;55:847–52. https://doi.org/10.1046/j.1365-2044.2000.01158.x

48. Estafanous FG, Brum JM, Ribeiro MP, Estafanous M, Starr N, Ferrario C. Analysis of heart rate variability to assess hemodynamic alterations following induction of anesthesia. J Cardiothorac Vasc Anesth. 1992;6:651–657. https://doi.org/10.1016/1053-0770(92)90045-9

49. Elwood T, Cecchin F, Low JI, Bradford HM, Goldstein B. Pilot study of preoperative heart rate variability and adverse events in children emerging from anesthesia. Pediatr Crit Care Med J Soc Crit Care Med World Fed Pediatr Intensive Crit Care Soc. 2005;6:54–7. https://doi.org/10.1097/01.PCC.0000149316.36372.2A

50. Ernst G, Watne LO, Frihagen F, Wyller TB, Dominik A, Rostrup M. Decreases in heart rate variability are associated with postoperative complications in hip fracture patients. PloS One. 2017;12:e0180423. https://doi.org/10.1371/journal.pone.0180423

51. Corrêa PR, Catai AM, Takakura IT, Machado MN, Godoy MF. [Heart rate variability and pulmonary infections after myocardial revascularization]. Arq Bras Cardiol. 2010;95:448–56. https://doi.org/10.1590/s0066-782x2010005000123

52. Powezka K, Adjei T, von Rosenberg W, Normahani P, Goverdovsky V, Standfield NJ, et al. A pilot study of preoperative heart rate variability predicting pain during local anesthetic varicose vein surgery. J Vasc Surg Venous Lymphat Disord. 2019;7:382–6. https://doi.org/10.1016/j.jvsv.2018.08.008

53. Adjei T, Von Rosenberg W, Goverdovsky V, Powezka K, Jaffer U, Mandic DP, et al. Pain Prediction from ECG in Vascular Surgery. IEEE J Transl Eng Health Med. T. Adjei, Department of Electrical and Electronic Engineering, Imperial College London, London, United Kingdom. E-mail: t.adjei15@imperial.ac.uk: Institute of Electrical and Electronics Engineers Inc.; 2017;5:8030035. https://doi.org/http://dx.doi.org/10.1109/JTEHM.2017.2734647

54. Nielsen R, Nikolajsen L, Krøner K, Mølgaard H, Vase L, Jensen TS, et al. Pre-operative baroreflex sensitivity and efferent cardiac parasympathetic activity are correlated with post-operative pain. Acta Anaesthesiol Scand. 2015;59:475–85. https://doi.org/10.1111/aas.12457

55. Mamode N, Docherty G, Lowe GD, Macfarlane PW, Martin W, Pollock JG, et al. The role of myocardial perfusion scanning, heart rate variability and D-dimers in predicting the risk of perioperative cardiac complications after peripheral vascular surgery. Eur J Vasc Endovasc Surg. 2001;22:499–508. https://doi.org/10.1053/ejvs.2001.1529

56. Hanss R, Block D, Bauer M, Ilies C, Magheli A, Schildberg-Schroth H, et al. Use of heart rate variability analysis to determine the risk of cardiac ischaemia in high-risk patients undergoing general anaesthesia. Anaesthesia. 2008;63:1167–73. https://doi.org/10.1111/j.1365-2044.2008.05602.x

57. May SM, Reyes A, Martir G, Reynolds J, Paredes LG, Karmali S, et al. Acquired loss of cardiac vagal activity is associated with myocardial injury in patients undergoing noncardiac surgery: prospective observational mechanistic cohort study. Br J Anaesth. 2019;123:758–67. https://doi.org/10.1016/j.bja.2019.08.003

58. Laitio TT, Huikuri HV, Mäkikallio TH, Jalonen J, Kentala ESH, Helenius H, et al. The breakdown of fractal heart rate dynamics predicts prolonged postoperative myocardial ischemia. Anesth Analg. 2004;98:1239–44.

59. Fleisher LA, Pincus SM, Rosenbaum SH. Approximate Entropy of Heart Rate as a Correlate of Postoperative Ventricular Dysfunction. Anesthesiology. 1993;78:683–92. https://doi.org/10.1097/00000542-199304000-00011

60. Marsch SCU, Skarvan K, Schaefer H-G, Naegeli B, Paganoni R, Castelli I, et al. Prolonged decrease in heart rate variability after elective hip arthroplasty. Br J Anaesth. 1994;72:643–9. https://doi.org/10.1093/bja/72.6.643

61. Kinoshita T, Asai T, Ishigaki T, Suzuki T, Kambara A, Matsubayashi K. Preoperative heart rate variability predicts atrial fibrillation after coronary bypass grafting. Ann Thorac Surg. 2011;91:1176–81. https://doi.org/10.1016/j.athoracsur.2010.12.042

62. Hakala T, Vanninen E, Hedman A, Hippeläinen M. Analysis of Heart Rate Variability does not Identify the Patients at Risk of Atrial Fibrillation after Coronary Artery Bypass Grafting. Scand Cardiovasc J. Taylor & Francis; 2002;36:167–71. https://doi.org/10.1080/cdv.36.3.167.171

63. Jideus L., Ericson M., Stridsberg M., Nilsson L., Blomstrom P. Diminished circadian variation in heart rate variability before surgery in patients developing postoperative atrial fibrillation. Scand Cardiovasc J. Norway: Informa Healthcare (69-77 Paul Street, London EC2A 4LQ, United Kingdom); 2001;35:238–44. https://doi.org/10.1080/14017430152581341

64. Ciszewski P, Tyczka J, Nadolski J, Roszak M, Dyszkiewicz W. Lower preoperative fluctuation of heart rate variability is an independent risk factor for postoperative atrial fibrillation in patients undergoing major pulmonary resection. Interact Cardiovasc Thorac Surg. 2013;17:680–6. https://doi.org/10.1093/icvts/ivt238

65. Kališnik JM, Hrovat E, Hrastovec A, Avbelj V, Žibert J, Geršak B. Severe Cardiac Autonomic Derangement and Altered Ventricular Repolarization Pave the Way to Postoperative Atrial Fibrillation. Innov Phila Pa. United States; 2015;10:398–405. https://doi.org/10.1097/IMI.0000000000000203

66. Vesela J, Osmancik P, Smrcka P, Bílek J, Herman D, Prochazkova R. PREOPERATIVE HEART RATE VARIABILITY ANALYSIS IN PATIENTS WITH NEW-ONSET ATRIAL FIBRILLATION AFTER CARDIAC SURGERY. Heart Rhythm. Netherlands: Elsevier B.V.; 2019;16:426–427. https://doi.org/http://dx.doi.org/10.1016/j.hrthm.2019.04.018

67. Chamchad D, Djaiani G, Jung HJ, Nakhamchik L, Carroll J, Horrow JC. Nonlinear heart rate variability analysis may predict atrial fibrillation after coronary artery bypass grafting. Anesth Analg. 2006;103:1109–12. https://doi.org/10.1213/01.ane.0000239330.45658.76

68. Chamchad D, Horrow JC, Samuels LE, Nakhamchik L. Heart rate variability measures poorly predict atrial fibrillation after off-pump coronary artery bypass grafting. J Clin Anesth. 2011;23:451–5. https://doi.org/10.1016/j.jclinane.2010.12.016

69. Tarkiainen TH, Hakala T, Hedman A, Vanninen E. Preoperative alterations in correlation properties and complexity of R-R interval dynamics predict the risk of atrial fibrillation after coronary artery bypass grafting in patients with preserved left ventricular function. J Cardiovasc Electrophysiol. 2008;19:907–12. https://doi.org/10.1111/j.1540-8167.2008.01139.x

70. Bari V, Ranucci M, De Maria B, Cairo B, Pistuddi V, Porta A. Model-based directional analysis of cardiovascular variability identifies patients developing atrial fibrillation after coronary artery bypass grafting. Int J Cardiol. 2018;258:97–102. https://doi.org/10.1016/j.ijcard.2018.01.071

71. Wu Z-K, Vikman S, Laurikka J, Pehkonen E, Iivainen T, Huikuri HV, et al. Nonlinear heart rate variability in CABG patients and the preconditioning effect. Eur J Cardiothorac Surg. 2005;28:109–13. https://doi.org/10.1016/j.ejcts.2005.03.011

72. Kališnik JM, Avbelj V, Vratanar J, Santarpino G, Geršak B, Fischlein T, et al. Cardiac autonomic regulation and PR interval determination for enhanced atrial fibrillation risk prediction after cardiac surgery. Int J Cardiol. 2019;289:24–9. https://doi.org/10.1016/j.ijcard.2019.04.070

73. Yum M-K, Oh A-Y, Lee H-M, Kim C-S, Kim S-D, Lee Y-S, et al. Identification of patients with childhood moyamoya diseases showing temporary hypertension after anesthesia by preoperative multifractal Hurst analysis of heart rate variability. J Neurosurg Anesthesiol. 2006;18:223–9. https://doi.org/10.1097/00008506-200610000-00001

74. Filipovic M, Jeger R, Probst C, Girard T, Pfisterer M, Gürke L, et al. Heart rate variability and cardiac troponin I are incremental and independent predictors of one-year all-cause mortality after major noncardiac surgery in patients at risk of coronary artery disease. J Am Coll Cardiol. 2003;42:1767–76. https://doi.org/10.1016/j.jacc.2003.05.008

75. Żebrowski JJ, Kowalik I, Orłowska-Baranowska E, Andrzejewska M, Baranowski R, Gierałtowski J. On the risk of aortic valve replacement surgery assessed by heart rate variability parameters. Physiol Meas. 2015;36:163–75. https://doi.org/10.1088/0967-3334/36/1/163

76. de Godoy MF, Takakura IT, Correa PR, Machado M de N, Miranda RC, Brandi AC. Preoperative nonlinear behavior in heart rate variability predicts morbidity and mortality after coronary artery bypass graft surgery. Med Sci Monit Int Med J Exp Clin Res. 2009;15:CR117-122.

77. Scheffler P, Muccio S, Egiziano G, Doonan RJ, Yu A, Carli F, et al. Heart rate variability exhibits complication-dependent changes postsurgery. Angiology. 2013;64:597–603. https://doi.org/10.1177/0003319712461932

78. Strous MTA, Daniels AM, Zimmermann FM, van Erning FN, Gidron Y, Vogelaar FJ. Is pre-operative heart rate variability a prognostic indicator for overall survival and cancer recurrence in patients with primary colorectal cancer? PloS One. 2020;15:e0237244. https://doi.org/10.1371/journal.pone.0237244

79. Ushiyama T, Mizushige K, Wakabayashi H, Nakatsu T, Ishimura K, Tsuboi Y, et al. Analysis of heart rate variability as an index of noncardiac surgical stress. Heart Vessels. 2008;23:53–9. https://doi.org/10.1007/s00380-007-0997-6

80. Bari V, Vaini E, Pistuddi V, Fantinato A, Cairo B, De Maria B, et al. Comparison of Causal and Non-causal Strategies for the Assessment of Baroreflex Sensitivity in Predicting Acute Kidney Dysfunction After Coronary Artery Bypass Grafting. Front Physiol. 2019;10:1319. https://doi.org/10.3389/fphys.2019.01319

81. Ferrario M, Moissl U, Garzotto F, Cruz DN, Tetta C, Signorini MG, et al. The Forgotten Role of Central Volume in Low Frequency Oscillations of Heart Rate Variability. PLOS ONE. Public Library of Science; 2015;10:e0120167. https://doi.org/10.1371/journal.pone.0120167

82. Yadav K, Singh A, Jaryal AK, Coshic P, Chatterjee K, Deepak KK. Modulation of cardiac autonomic tone in non-hypotensive hypovolemia during blood donation. J Clin Monit Comput. 2017;31:739–46. https://doi.org/10.1007/s10877-016-9912-y

83. Kinsella SM, Carvalho B, Dyer RA, Fernando R, McDonnell N, Mercier FJ, et al. International consensus statement on the management of hypotension with vasopressors during caesarean section under spinal anaesthesia. Anaesthesia. 2018;73:71–92. https://doi.org/10.1111/anae.14080

84. Riznyk L, Fijałkowska M, Przesmycki K. Effects of thiopental and propofol on heart rate variability during fentanyl-based induction of general anesthesia. Pharmacol Rep PR. 2005;57:128–34.

85. Francis DP, Willson K, Georgiadou P, Wensel R, Davies LC, Coats A, et al. Physiological basis of fractal complexity properties of heart rate variability in man. J Physiol. 2002;542:619–29. https://doi.org/10.1113/jphysiol.2001.013389

86. van den Berg MP, Hassink RJ, Baljé-Volkers C, Crijns HJGM. Role of the autonomic nervous system in vagal atrial fibrillation. Heart Br Card Soc. 2003;89:333–5. https://doi.org/10.1136/heart.89.3.333

87. Bilchick KC, Berger RD. Heart Rate Variability. J Cardiovasc Electrophysiol. 2006;17:691–4. https://doi.org/10.1111/j.1540-8167.2006.00501.x

88. Malpas SC, Maling TJB. Heart-Rate Variability and Cardiac Autonomic Function in Diabetes. Diabetes. American Diabetes Association; 1990;39:1177–81. https://doi.org/10.2337/diab.39.10.1177

89. Kehlet H. Enhanced postoperative recovery: good from afar, but far from good? Anaesthesia. 2020;75:e54–61. https://doi.org/https://doi.org/10.1111/anae.14860
